# Comparison of polymerase chain reaction and microscopy for the detection of *Fasciola* spp. in the fecal matter of domestic bovines in Kalasin Province, Thailand

**DOI:** 10.14202/vetworld.2021.2878-2882

**Published:** 2021-11-10

**Authors:** Sirikanda Thanasuwan, Anupong Tankrathok

**Affiliations:** 1 Department of Veterinary Technology, Faculty of Agricultural Technology, Kalasin University, Kalasin, Thailand; 2 Department of Biotechnology, Faculty of Agricultural Technology, Kalasin University, Kalasin, Thailand

**Keywords:** *28s rRNA*, bovine, *Fasciola gigantica*, Kalasin province, Lampao dam, polymerase chain reaction

## Abstract

**Background and Aim:**

*Fasciola* spp. are important foodborne trematodes and waterborne zoonotic parasites that cause health problems and economic losses worldwide, including in Thailand. *Fasciola* spp. are usually detected by sedimentation or the formalin-ethyl acetate concentration technique (FECT) under microscopy, which is less specific and sensitive. Accurate detection is important to detect real incidence for protection against and elimination of fasciolosis in the area. This study aimed to determine the distribution of *Fasciola* spp. and compare the specificity and sensitivity of FECT under microscopy to that of polymerase chain reaction (PCR) in cattle feces.

**Materials and Methods:**

The study was conducted in Kalasin Province, Thailand. Feces of 46 cattle were investigated for infection with *Fasciola* spp. To detect infection, FECT under microscopy and PCR amplification of the *28S rRNA* gene of *Fasciola* spp. were used to identify egg parasites.

**Results:**

Feces of 16 of 46 (34.78%) cattle were positive for *Fasciola* spp. using FECT under microscopy, whereas PCR showed that 67.39% (31 of 46) were positive for *Fasciola* spp. False-negative results were as high as 32.61% when diagnosed under microscopy.

**Conclusion:**

This study confirmed the infection of cattle with *Fasciola* spp. in Kalasin Province, indicating that PCR demonstrated higher sensitivity and specificity when diagnosing infection. FECT under microscopy can still be used as a primary and traditional method for diagnosis. However, relapse cases of *Fasciola* spp. and *Paramphistomum* spp. should be diagnosed by microscopy combined with PCR. This is the first report on the molecular distribution of fecal samples in cattle in Kalasin Province.

## Introduction

*Fasciola* spp. are major foodborne trematodes and waterborne zoonotic parasites that cause health problems and economic losses worldwide [1]. Fasciolosis may also occasionally affect humans. The lifecycle of *Fasciola* spp. requires two hosts. Cattle are a definitive host, and the snail is an intermediate host. A human is an accidental host [2]. It is an important public health threat due to foodborne trematode zoonoses in many countries, including Thailand [3,4]. The prevalence is high in areas surrounding dams or large areas of water in which *Lymnaea* spp., the intermediate host of *Fasciola* spp., is found [5]. These parasites can infect >40 species of wild and domesticated animals (e.g., bovines, buffaloes, sheep, and goats) [6].

In animals, *Fasciola* spp. have a significant impact on growth rate, development, reduction of meat production and milk, decreased fertilization [7], occurrence of chronic diseases and anemia, lethargy, weight loss, and lower fertility rates [8], liver spoilage, high morbidity rates, and wool in livestock ruminants [9]. Infection occurs when animals ingest the parasites while grazing or drinking water contaminated with metacercaria. The juvenile worm then moves through the liver parenchyma; finally, the fluke will reside in the bile duct. In developed countries, the occurrence of *Fasciola* spp. can be as high as 77% [2]. In general, the determination of fasciolosis in ruminants caused by *Fasciola* spp. has been made only by examining *Fasciola* eggs in feces. In Thailand, the occurrence rate of fasciolosis in cattle and buffaloes ranges between 4% and 24% in feces when investigated under a microscope [10-15]. Infections are most prevalent in northeast Thailand, where infection rates in cattle and buffaloes can be as high as 85% [16]. However, only one report has detected *Fasciola* spp. in feces using polymerase chain reaction (PCR) [17].

*Fasciola* spp. are usually detected by sedimentation or formalin-ethyl acetate concentration technique (FECT) under microscopy, which is easy and inexpensive in terms of reagents and equipment but less sensitive in case of a low percentage of parasites and less specific in the case of an unexperienced microscopist [18]. Furthermore, certain parasites, such as *Fasciola* spp. and *Paramphistomum* spp., could be mistaken for each other, as their eggs are morphologically very similar. This may lead to misdiagnosis and subsequent application of the wrong treatment [13]. Although FECT has been used to concentrate the eggs in feces to increase the probability of detecting egg parasites [19], the phase where eggs can be found, from infection to adult worm (prepatent period [PPP]), takes between 13 and 14 weeks after infection. Adult worms are unable to produce eggs before the PPP has been completed. Therefore, eggs in the stool cannot be found before the PPP has been completed [18-20]. Molecular diagnosis by PCR can reduce the limitations of microscopic diagnosis of fasciolosis [21]. In addition, PCR can report the results of the current infection [16].

This study was conducted to determine the prevalence of *Fasciola* spp. in cattle feces and compare the specificity and sensitivity of microscopy to conventional PCR in diagnosing fasciolosis.

## Materials and Methods

### Ethical approval

This study was approved by the Institutional Ethical Committee of Kalasin University, Thailand. Samples were collected without any harm to the cattle and in accordance with standard procedures.

### Study period, area, and sample collection

The study was conducted during October and November 2020. In this study, adult flukes were collected from livers of naturally infected cattle from Kalasin province, Thailand. The samples were transported under low-temperature conditions (<10°C) to the Laboratory of the Department of Veterinary Technology, Faculty of Agricultural Technology, Kalasin University. The parasites were washed in normal saline and stored at −20°C until used for molecular identification.

Fecal samples of cattle were collected randomly from different locations in Kalasin during October and November 2020. Forty-six fresh fecal samples were collected directly from the rectum of cattle by hand using plastic gloves. The individual fecal samples were brought to the laboratory and processed in the Technology Veterinary Laboratory of Kalasin University, Thailand. The samples were stored at 4°C until the use.

### FECT

Feces (2 g) were added to normal saline (15-20 mL), and the dissolving feces were mixed by vortexing. Each dilution was filtered through a wet gauze inside a funnel into a 15 mL glass conical centrifuge tube and centrifuged at ~1500 rpm for 3 min. The supernatant was discarded, and 7 mL of a 10% formalin solution were added and mixed well. Ethyl acetate (3 mL) was added, and the tube was shaken vigorously for 1 min. The tube was centrifuged at ~1500 rpm for 3 min. The results showed three layers at the top of the tube: A top layer of ether, a plug of fecal debris and formalin, and sediment of egg parasites at the bottom. Next, a stick was used to loosen the layer of fecal debris from the side of the tube, and the ether was discarded. The remaining sediment was mixed by autopipetting, and one drop was added to a drop of saline on a glass slide. The sediment was covered with a coverslip and examined microscopically using 10× and 40× objectives for the presence of *Fasciola* egg forms [21].

### DNA extraction

Genomic DNA was extracted from part of adult flukes using a GF-1 Tissue DNA Extraction Kit (Vivantis, Malaysia) according to the manufacturer’s instructions. Genomic DNA was eluted in 50 μL elution buffer and kept at −20°C until used for molecular identification.

DNA was extracted from 2 mg feces (n=46 samples) using a QIAamp^®^ DNA Stool Mini Kit (Qiagen, Hilden, Germany) according to the manufacturer’s protocol. Genomic DNA was eluted in 50 μl elution buffer and stored at −20°C until used for molecular identification.

### PCR

A fragment of 618 bp of the *28S rRNA* gene of *Fasciola* spp. was amplified using two primers (forward: 5’-ACGTGATTACCCGCTGAACT-3′ and reverse: 5’-CTGAGAAAGTGCACT GACAAG-3’) [1,22,23]. PCR was performed with 25 μL reaction buffer containing 2 μL genomic DNA (10-100 ng), 1 U Taq DNA polymerase (Vivantis), 0.2 mM deoxynucleotide triphosphates, 1.75 mM MgCl_2_, 2.5 μL PCR buffer (10×), and 0.5 μM of each primer. The reaction was performed in a T100 Thermal Cycler (Bio-Rad, USA) with the following PCR conditions: Initial DNA denaturation at 94°C for 4 min, 30 cycles of DNA denaturation at 94°C for 45 s, primer annealing at 58°C for 45 s, primer extension at 72°C for 45 s, and extension step at 72°C for 10 min. PCR products were analyzed by electrophoresis on a 1.5% agarose gel for ~45 min at 100 V and visualized by staining with 1% ethidium bromide.

## Results

### Microscopy and PCR prevalence rates

**Figure-1:**
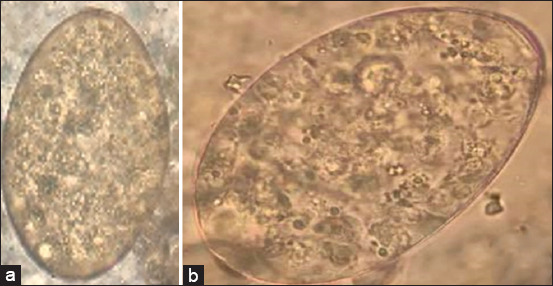
*Fasciola* egg present in fecal of cattle under microscopy 40× (a) *Fasciola* spp. egg (b) *Paramphistomum* spp. egg.

**Figure-2:**
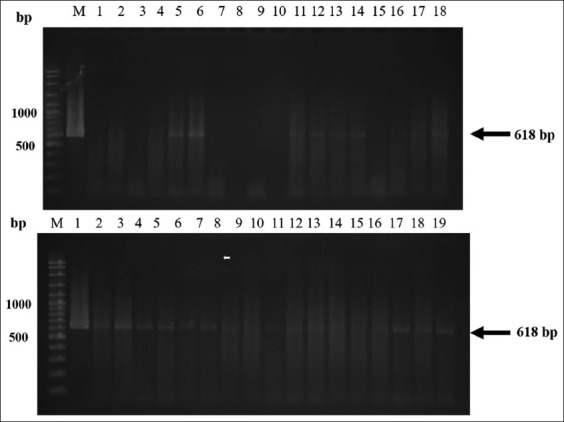
Polymerase chain reaction product 618 bp of partial mitochondria *28s rRNA* gene. Lane M: DNA ladder (100 bp). Lane 1: Positive control (adult *Fasciola* spp.), Lane: 2-18 represent different samples of *Fasciola* egg.

Forty-six fecal samples under microscopy showed large brown eggs with an operculum identified as *Fasciola* sp. eggs according to their morphology. Only 34.78% (16 of 46) of the fecal samples were positive for *Fasciola* spp. infection using FECT detection under a microscope. *Fasciola* spp. and *Paramphistomum* spp. eggs were morphologically very similar, leading to misdiagnosis and subsequent treatment failure (Figure-1). Therefore, molecular diagnosis was used for identification based on partial nucleotide sequences of the mitochondrial *28S rRN*A gene. The proliferation of DNA fragments of *Fasciola* spp., at a total length of 618 bp DNA, was determined by applying a specific primer to the egg, *Fasciola* spp. The adult parasite was used as a positive control (test result comparison; Figure-2). PCR showed that 67.39% (31 of 46) of the fecal samples were positive, and false-negative results were as high as 32.61% when diagnosed under microscopy (Table-1).

**Table-1:** Comparison between microscopy and PCR techniques for examining the prevalence of *Fasciola* spp. in beef cattle.

Parasites/techniques	Microscopy		PCR		
	
	% Positive (n)	% Negative (n)	% Positive (n)	% Negative (n)	% False-negative
*Fasciola* spp.	34.78 (16/46)	65.22 (30/46)	67.39 (31/46)	32.61 (15/46)	32.61 (15/46)

PCR=Polymerase chain reaction

## Discussion

Fasciolosis is an important food-and water-borne parasitic zoonosis that impact livestock production development in many parts of the world, including Thailand, and may have negative economic consequences [13]. Several studies in Thailand have shown that *Fasciola* spp. are endemic in cattle, buffaloes, and humans [3,10,13,14,24-27]. Microscopic detection by sedimentation and FECT is practical and routinely used to diagnose *Fasciola* spp. in feces, but this method has problems and limitations related to microscopic diagnoses, such as the lack of skilled microscopists, variation in training or experience, unsuitable microscopes, variations in reagents in the apparatus, and insufficient quality control [18]. In Thailand, there has been only one molecular diagnosis (PCR) reported case of feces of ruminants infected with this parasite [17]. This is the first molecular diagnosis report about the prevalence of *Fasciola* spp. in cattle feces in Kalasin Province. However, many authors have reported a 5-20% prevalence rate of *Fasciola* spp. under microscopic investigation [12,13]. Eggs of flukes were identified using keys, as described previously by Soulsby [28]. In this study, molecular diagnostic assays, such as PCR, were used for genus identification and confirmation of the results. This is a more dependable, sensitive, and precise detection method. In this study, the higher infection rate (67.39%) of fasciolosis by PCR could be compared to the low infection rate by microscopy (34.78%). The cause of a low infection rate by the microscopic assay could be that early infection was not found as the lifecycle of this parasite is ~12 weeks. Infection in fecal samples was detected under microscopy, but this technique has limited sensitivity for animals infected with *Fasciola* spp. and is only detected after the PPP has passed [19]. Conventional PCR tests can detect the pathology early in the infection stage where DNA of *Fasciola* spp. can be found in feces samples before the lifecycle is complete. Experiments have reported that DNA of the parasites was detected before the completion of the PPP (<8 weeks) [29]. The cause of finding parasite DNA in the fecal matter, before finding fluke eggs were found in the fecal matter, could be from cell fragments of larval parasites, such as “free flukes.” The part of the parasite released is not DNA from eggs but DNA from dead skin cells of both larval and adult parasites that respond to the animal’s immune response [19].

Therefore, molecular guidelines, such as PCR, have greatly increased the ability to differentiate members of the genus *Fasciola* and thus show the importance of molecular techniques for identifying and differentiating *Fasciola* spp. Moreover, in this study, false-negative results were as high as 32.61% when diagnosed under microscopy. This may be because *Fasciola* spp. and *Paramphistomum* spp. eggs are identical and have a similar structure, which may result in a misdiagnosis [8]. Diagnosis by microscopy of cattle feces is an easy and economical technique. However, this technique is less sensitive than PCR. In addition, another limitation of microscopy is that it cannot clearly differentiate between different species of *Fasciola*, whereas the molecular technique can differentiate them [18].

This study showed similar results to others that applied *28S rRNA* primers to detect and diagnose *Fasciola* spp. from naturally infected ruminants [1,22,23] and cercariae in snails in Thailand. The results showed that the molecular method (PCR) is the most sensitive assay for examining and identifying *Fasciola* infections [30]. The examination of *Fasciola* eggs to the species level normally depends on size, shape, and color differences. However, this result has indicated that the microscopy method to be unreliable. *Fasciola* spp. eggs and rumen flukes are similar, but they can be distinguished by morphology together with methylene blue staining [13] or using the method of Parfitt and Banks to differentiate eggs of flukes [31]. However, the prevalence of *Fasciola* spp. in cattle feces around Phayao Lake, Phayao, Thailand, showed only 8.4% of infection with *Fasciola* spp. using methylene blue staining [13]. This method requires specialist skills and expertise to detect infections. Fasciolosis is a foodborne zoonotic trematode, and more than 25 cases of human infections have been reported in Thailand between 1990 and 2006 [32]. It is very important to apply PCR for epidemiological study for human and animal fasciolosis in Thailand.

## Conclusion

Conventional PCR for diagnosis of *Fasciola* spp. in fecal matter is specific and sensitive. The test is suitable for the study of the prevalence and follow-up for drug treatment in cattle. It would be useful for fasciolosis control programs, and PCR needs to be applied in endemic areas using stool DNA extraction methods.

## Authors’ Contributions

ST: Planned and designed the experiment, analyzed the data, and conducted the fieldwork. AT: Analyzed the data and revised the manuscript. Both authors have read and approved the final manuscript.
